# Interrogating accessibility of telomeric sequences with FRET-PAINT: evidence for length-dependent telomere compaction

**DOI:** 10.1093/nar/gkab067

**Published:** 2021-03-10

**Authors:** Golam Mustafa, Sajad Shiekh, Keshav GC, Sanjaya Abeysirigunawardena, Hamza Balci

**Affiliations:** Department of Physics, Kent State University, Kent, OH 44242, USA; Department of Physics, Kent State University, Kent, OH 44242, USA; Department of Chemistry and Biochemistry, Kent State University, Kent, OH 44242, USA; Department of Chemistry and Biochemistry, Kent State University, Kent, OH 44242, USA; Department of Physics, Kent State University, Kent, OH 44242, USA

## Abstract

Single-stranded telomeric overhangs are ∼200 nucleotides long and can form tandem G-quadruplex (GQ) structures, which reduce their accessibility to nucleases and proteins that activate DNA damage response. Whether these tandem GQs further stack to form compact superstructures, which may provide better protection for longer telomeres, is not known. We report single-molecule measurements where the accessibility of 24–144 nucleotide long human telomeric DNA molecules is interrogated by a short PNA molecule that is complementary to a single GGGTTA repeat, as implemented in the FRET-PAINT method. Binding of the PNA strand to available GGGTTA sequences results in discrete FRET bursts which were analyzed in terms of their dwell times, binding frequencies, and topographic distributions. The binding frequencies were greater for binding to intermediate regions of telomeric DNA compared to 3′- or 5′-ends, suggesting these regions are more accessible. Significantly, the binding frequency per telomeric repeat monotonically decreased with increasing telomere length. These results are consistent with telomeres forming more compact structures at longer lengths, reducing accessibility of these critical genomic sites.

## INTRODUCTION

Human telomeres are composed of long tracts of hexanucleotide d(GGGTTA) repeats that end with a 3′ single-stranded overhang, which is several hundred nucleotide (nt) long (1–5). After each round of replication, telomeres are shortened until senescence or apoptosis is triggered when the overhang length shortens to ∼50 nt (6). Telomerase, a reverse transcriptase, elongates telomeres by adding repeat segments of d(GGGTTA) to the 3′-end using the RNA template TERRA. Telomerase is highly expressed in most cancer cells (7–9), whereas in normal cells its activity is very low (10). Four repeats of d(GGGTTA) sequence form a G-quadruplex (GQ) structure *in vitro* (11,12) and *in vivo* (13–17). Stacked co-planar tetrads, stabilized by Hoogsteen-type base pairing and monovalent cations, are characteristics of the GQ structure (18–20). GQ structures are very stable and are presumed to protect the otherwise vulnerable telomeric overhangs against enzymatic activity by reducing their accessibility (21). Human telomeric GQs inhibit telomerase activity by rendering telomeric ends inaccessible to telomerase-mediated extension (22–24). This potential, coupled with their distinct planar structure, has made them an attractive target for anti-cancer drugs that stabilize the GQ against telomerase activity (25). Therefore, study of accessibility of human telomeric overhangs and folding characteristics of telomeric GQs have considerable significance.

Numerous studies have reported on the relationship between solution conditions and the stability, topology, and folding pathways of single GQ molecules (20,24,26–30) and their interactions with GQ-stabilizing small molecules (31). Their stability against ssDNA binding proteins (32,33) and helicases (34–36) has also been investigated. However, the telomeric overhangs are long enough to accommodate ∼10 tandem GQs, which might form higher order structures that impact the overall accessibility of telomeres (37–39). Advances in synthesizing high-purity telomeric repeats, significantly longer than the commonly used four d(GGGTTA) repeats, have enabled expanding biophysical studies to sequences that better mimic telomeric overhangs. Earlier works (40–47) have focused on understanding the structures formed by multiple GQs, the potential interactions between them, and how such interactions impact the overall compactness and stability. However, a consensus has not yet emerged about these questions. Different studies have found negligible interactions, stabilizing stacking interactions, or frustrated folding and destabilizing interactions due to neighboring folded GQs, i.e. negative cooperativity. A summary of these studies is given below. For brevity, the d(GGGTTA) sequence will be referred to as a ‘G-Tract’ in this summary and the rest of the manuscript.

Using biophysical assays, Yu *et al.* concluded that telomeric sequences that contain 8–16 G-Tracts form consecutive tandem GQs connected with TTA linkers, like beads-on-a-string, with negligible stacking interactions between the GQs (40). Using similar methods and molecular dynamics simulations, Petraccone *et al.* suggested that sequences containing 8–12 G-Tracts formed higher-order structures with two or three contiguous GQs, respectively (41). Two atomic force microscopy studies on a 96 nt long telomeric sequence yielded different results where Xu *et al.* reported formation of a compact structure of four tandem GQs (42), while Wang *et al.* reported only two tandem GQs (43). In an optical tweezers study, Punnoose *et al.* reported that GQs formed randomly in long telomere overhangs within seconds, but these GQs were separated from each other by unstructured G-Tracts (44). This observation was justified by the folding process being dominated by the kinetics rather than thermodynamics. Using electron microscopy, Kar *et al.* showed that very long (up to 20 000 nt) human telomeric sequences within single stranded DNA (ssDNA) can spontaneously condense into chains of large discrete bead-like particles of two different sizes, compacting the telomeric DNA nearly 12-fold in length (45). In an NMR study, Sannohe *et al.* proposed formation of rod-like higher-order structures in ^Br^G-substituted human telomeric oligonucleotides (46). In another biophysical study, Vorlíčková *et al.* reported that the thermal stability of GQs decreases with increasing G-Tract number for 1–17 G-Tract long telomeric sequences, suggesting destabilizing interactions between tandem GQs (47). Considering the sensitivity of the conformation and stability of even a single telomeric GQ to annealing, storage and ionic conditions (48), we believe some of the discrepancies among these studies are due to factors associated with sample preparation and assay conditions.

In this study, we investigated how accessibility of telomeres varies as a function of sequence length and position of target sites within the telomere. Telomeric DNA sequences containing 4–24 G-Tracts (24–144 nt), which can form 1–6 tandem GQs, were examined at the single molecule level using the FRET-PAINT technique (49,50) that combines DNA-PAINT (51) (Point Accumulation for Imaging in Nanoscale Topography) with single molecule Förster resonance energy transfer (smFRET) spectroscopy. DNA-PAINT has been used as a super-resolution technique as it allows fluorescently labeled DNA probes to transiently bind to different regions of a system and allow localization of the binding sites with high accuracy. By accumulating large sets of such binding and localization events, distributed over time, it has been possible to construct structures beyond the diffraction limit. The telomeric sequences that were investigated in this study also have multiple binding sites (G-Tracts) that are distributed over the telomeric overhang and are not necessarily static. Therefore, interrogating such sites with probes that transiently bind has the potential to reveal details of the underlying structures. However, telomeric sequences form compact structures that have characteristic lengths in 1–10 nm range, which is challenging to resolve even for super-resolution microscopy. On the other hand, this is the ideal range for smFRET, which motivated us to synthesize elements of DNA-PAINT, such as transient binding of fluorescently labeled nucleic acids, with smFRET spectroscopy, which enables resolving sub-nanometer distances in the length scale of interest.

**Figure 1.**
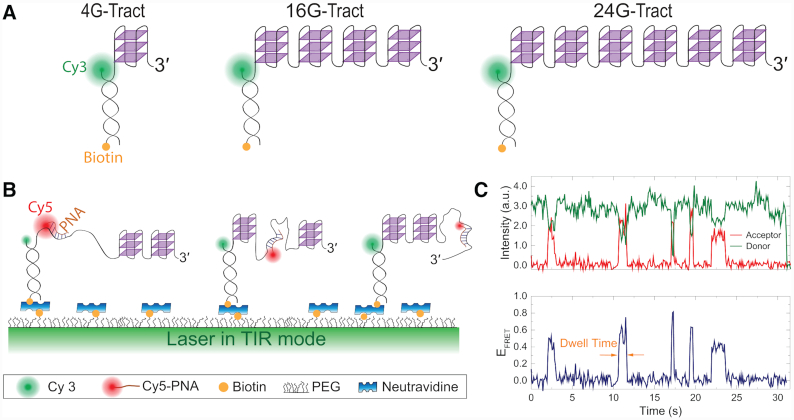
Schematic of DNA constructs and an example smFRET trace. (**A**) Partial duplex DNA constructs were formed by a biotinylated short strand (labeled with donor fluorophore Cy3) and a longer strand that contains a telomeric overhang with 4–24 repeats of GGGTTA sequence. For brevity, only three of the six studied constructs are shown. The 4G-Tract, 16G-Tract and 24G-Tract constructs can fold into a maximum of one, four and six tandem GQs, respectively. (**B**) Schematics of smFRET channel surface where the pdDNA constructs are immobilized on the PEGylated surface via biotin-neutravidin attachment. The donor fluorophore is excited in TIR mode with a 532-nm laser beam. The PNA strand, which is labeled with the acceptor fluorophore (Cy5), is complementary to a G-Tract and binds to available (unfolded) G-Tracts for a short period of time before dissociating. These binding events result in bursts in acceptor intensity and FRET efficiency. (**C**) Example smFRET time trace that shows five binding events to a single telomeric overhang. The dwell time for one of these binding events is indicated by orange arrows. Binding to different segments of the overhang results in different FRET levels.

In our approach, a short Peptide Nucleic Acid (PNA) strand, which is complementary to a single G-Tract and fluorescently labeled with an acceptor fluorophore (Cy5), is introduced to a microfluidic channel that contains surface-immobilized partial duplex DNA (pdDNA) constructs. The pdDNA constructs are labeled with a donor fluorophore (Cy3) and contain telomeric sequences at their overhangs (Figure 1A and B). The surface-immobilized Cy3-pdDNA serves as the docking construct and Cy5-PNA as the imager strand. The Cy5-PNA molecule transiently binds to G-Tracts that are not part of a GQ, resulting in a FRET signal that varies depending on how far the Cy5-PNA binds from the Cy3. By using low-enough Cy5-PNA concentration, each binding and dissociation event can be detected in isolation from other events. We determined how the frequency, dwell-time and topography of binding events vary for six different DNA constructs that can form 1–6 GQs. For each of these constructs, we also characterized how dwell-time and binding frequency vary for different segments of the sequence, e.g. 3′ end, middle section, or 5′-end.

## MATERIALS AND METHODS

### DNA constructs

DNA and PNA sequences are given in Supplementary Table S1. The pdDNA constructs were made by annealing a short (18 nt) stem strand, with biotin at 3′-end and Cy3 at 5′-end, and a long strand that contains a complementary sequence to the stem and 4–24 G-Tracts. The two strands were heated to 95°C for 3 min, followed by cooling to 30°C with a temperature decrease step of 5°C, and waiting for 3 min in each step in a thermal cycler (Hybaid Omn-E Thermal Cycler). The annealing reaction was performed at 150 mM KCl and 2 mM MgCl_2_ (except for certain data sets in Supplementary Figures S2 and S3 where 150 mM LiCl and 2 mM MgCl_2_ were used).

The long strands with 8–24 G-Tracts were purchased from Eurofins Genomics (Louisville, KY, USA) without additional purification, while the unpurified 4G-Tract and the HPLC purified stem strand were purchased from Integrated DNA Technologies (Coralville, IA, USA). All the unpurified strands were purified in-house using denaturing polyacrylamide gel electrophoresis (PAGE). Supplementary Figure S1 shows the radiographs of denaturing PAGE for purified DNA oligonucleotides radiolabeled with Phosphorus-32 isotope. The HPLC purified short Cy5-PNA docking strand was purchased from PNA Bio (Thousand Oaks, CA, USA). The schematics of pd-4G-Tract, pd-16G-Tract, and pd-24G-Tract constructs are shown in Figure 1A, where anti-parallel GQ conformation and maximum number of GQ folding were assumed.

### Sample preparation and smFRET assay

Laser-drilled quartz slides and glass coverslips were extensively cleaned with potassium hydroxide (KOH) and acetone, followed by piranha etching, aminosilane coating and NHS-ester polyethylene glycol (PEG) passivation. A PEG mixture with 100:2 ratio of m-PEG-5000: biotin-PEG-5000 (Laysan Bio Inc.) was used for surface passivation. PEG prohibits non-specific binding of DNA and PNA molecules to the surface, whereas biotin-PEG-5000 offers an attachment point for molecules of biotinylated DNA that connect to the biotin-PEG through neutravidin. An additional round of PEGylation was done using a small (333 Da) MS(PEG)_4_ (purchased from Thermofisher Scientific) to densify the PEG layer. The sample chambers were prepared by sandwiching double-sided tape between a slide and coverslip, both of which were PEGylated. The chamber was treated with 1% (v/v) Tween-20 (15 min incubation) followed by extensive washing and introduction of 0.01 mg/ml neutravidin.

The pdDNA samples were diluted to ∼40 pM in multiple steps and incubated in the sample chamber for 2–5 min at 150 mM KCl (except for certain datasets in Supplementary Figures S2 and S3 where 150 mM LiCl was used) and 2 mM MgCl_2_, which is also maintained during all measurements. The chambers were then washed with imaging buffer to remove excess DNA. This protocol yielded 300–330 molecules to be immobilized per imaging area of ∼50 × 100 μm^2^. The imaging buffer contained Tris base (50 mM, pH 7.5), 2 mM Trolox, 0.8 mg/ml glucose, 0.1 mg/ml glucose oxidase, 0.1 mg/ml bovine serum albumin (BSA), 2 mM MgCl_2_ and 150 mM KCl (except for certain datasets in Supplementary Figures S2 and S3 where 150 mM LiCl was used instead of 150 mM KCl) and 40 nM Cy5-PNA. Cy5-PNA strand stock solution was heated for 10 min at 85°C to increase solubility. After removing it from the heat bath, the tube that contained PNA was left at room temperature for 15 min before it was diluted 100-fold in imaging solution. The imaging solution that contained Cy5-PNA was incubated with the DNA molecules in the channel for 15 min prior to video recording. Movies of 2000 frames were recorded at a frame integration time of 100 ms/frame.

### FRET-PAINT

A schematics of FRET-PAINT assay is presented in Figure 1B. Under 532-nm laser beam excitation, Cy3-labeled, surface-immobilized pdDNA samples yield almost zero FRET unless a Cy5-PNA strand binds to a G-Tract at the overhang. Bursts in acceptor intensity and FRET efficiency occur when the Cy5-PNA binds to a G-Tract.

### Imaging setup

The measurements were carried out using a prism-type total internal reflection (TIR) fluorescence setup equipped with an Olympus IX-71 microscope and an Andor IXON EMCCD camera (IXON DV-887 EMCCD, Andor Technology, CT, USA-now part of Oxford Instruments). The IXON camera has 512 × 512 pixels and a pixel size of 16 μm. The donor fluorophore was excited with 532-nm laser beam (Spectra Physics Excelsior). An Olympus water objective (60×, 1.20 NA) was used to collect the fluorescence signal. The total magnification in our setup is 90x which results in an effective pixel size of 178 nm. The laser beam intensity was approximately 1 kW/cm^2^.

### Data analysis

**Figure 2.**
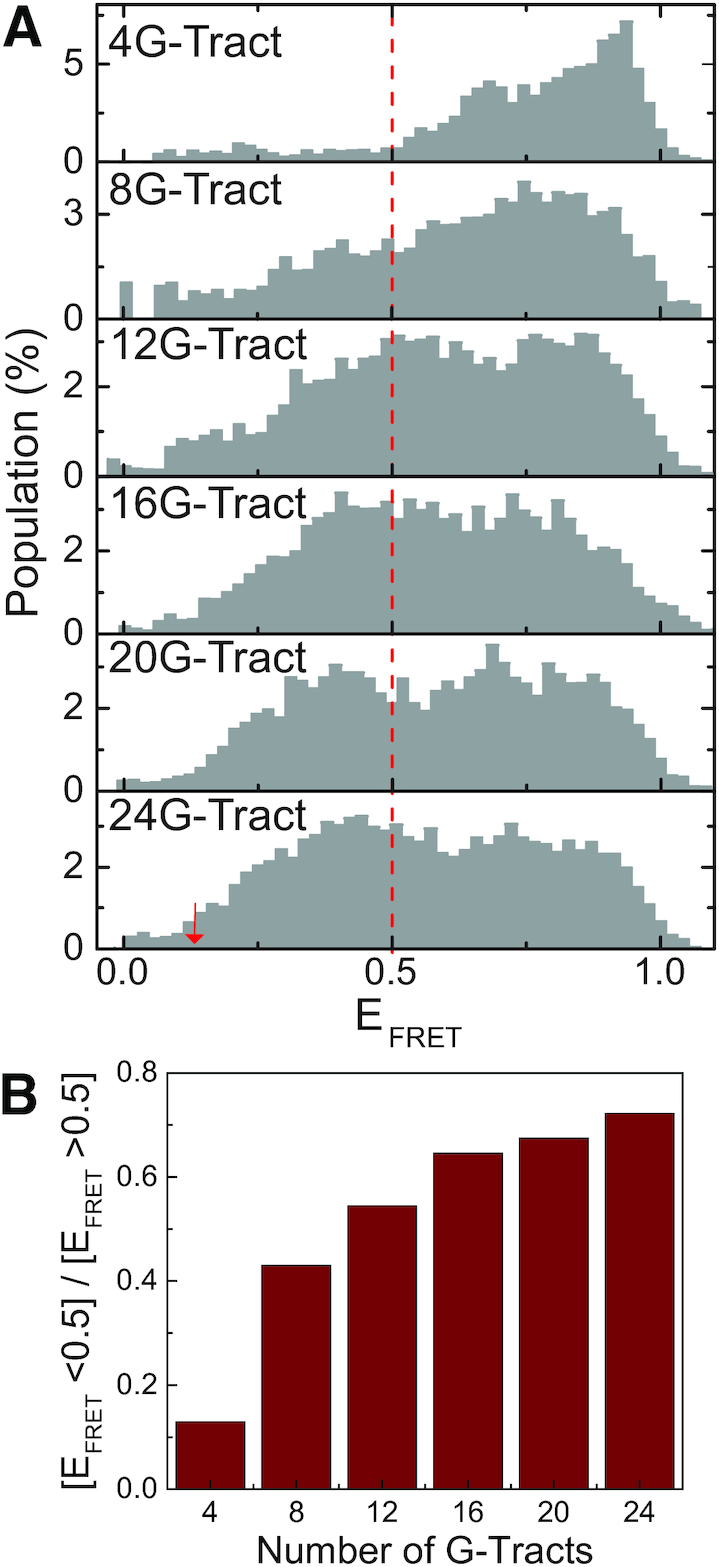
Normalized FRET histograms based on FRET-PAINT. (**A**) Each normalized FRET efficiency histogram is constructed from hundreds of short PNA binding events as exemplified in Figure 1C (exact numbers are given in Figure 3 caption where these events are analyzed in terms of their dwell times). As the number of G-Tracts increases, the population of lower FRET efficiency states increases since binding sites further from the donor become available. The number of molecules (telomeric overhangs that had at least one binding event) in each histogram is *N* = 127, 170, 210, 260, 185 and 230 for the 4G-, 8G-, 12G-, 16G-, 20G- and 24G-Tract construct, respectively. The red arrow in 24G-Tract construct indicates the E_FRET_ = 0.13 level which is used as a threshold in Figure 3. (**B**) The ratio of the total population of [*E*_FRET_ < 0.50] to [*E*_FRET_> 0.50] states is plotted. As expected, this ratio increases as the number of G-Tracts increases.

The recorded movies were analyzed using custom software written in C++ to generate time traces of donor intensity (*I*_D_) and acceptor intensity (*I*_A_) for each molecule. Using a custom MATLAB code, the time traces were screened to ensure they represent single molecules. The background was subtracted from each of these selected molecules, which were then used to determine dwell time, binding frequency, and FRET distributions presented in this manuscript. The FRET efficiency (*E*_FRET_) was calculated using *E*_FRET_*= I*_A_*/*(*I*_A_ *+ I*_D_). E_FRET_ population histograms (Figure 2A) were constructed from screened single molecule traces that showed at least one Cy5-PNA strand binding event. The contribution of molecules was normalized such that each molecule contributed equally to the histogram, and the total population of the histogram was normalized to 100%. The screened single molecules that did not show any binding event contributed to a donor-only (DO) peak, which is due to leakage of donor emission into acceptor channel. The DO peak was used as reference to shift the DO *E*_FRET_ to zero and rescale the FRET range.

**Figure 3.**
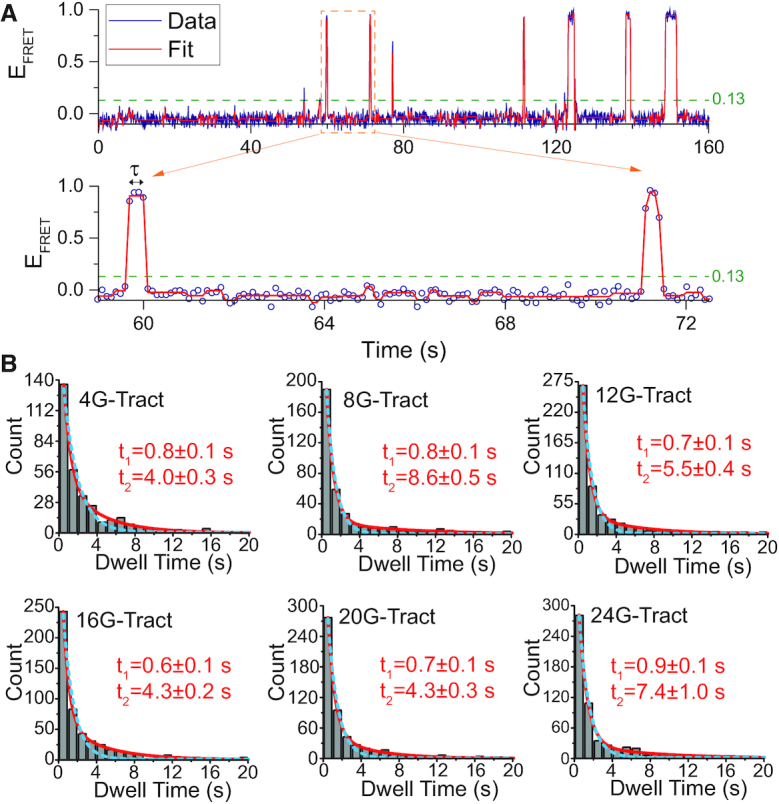
Determining the characteristic dwell times. (**A**) An example smFRET time trace that shows seven PNA binding events to a single telomeric overhang. A *Stepfinder* fit (red line) is used to characterize the FRET levels and dwell times (τ) of the binding events. The dashed line at *E*_FRET_ = 0.13 represents the threshold level used for considering a FRET burst a PNA binding event, rather than noise. The signal bursts should also satisfy τ≥300 ms to be considered a binding event. (**B**) Distribution of dwell times determined from *Stepfinder* fit for each construct. The distributions were better described with a double exponential (red line) fit compared to a single exponential (dashed cyan line) fit, which consistently underestimated the counts of events with longer dwell times. In the double exponential, ${t_1}$ (faster decay) is the characteristic time for short dwells and ${t_2}$ (slower decay) is the characteristic time for long dwells. The number of binding events in the histograms for 4G-, 8G-, 12G-, 16G-, 20G- and 24G-Tract constructs is N = 331, 394, 561, 556, 563 and 604, respectively.

The *E*_FRET_ levels and dwell times (τ) of the binding events in smFRET time traces were characterized by an automated and bias-free step-detection method, *Stepfinder* (52), that can determine steps in large datasets without requiring prior knowledge of their distribution. It starts by fitting a single step to the data with a location and size which gives the lowest residual chi-square (53). Figure 3A shows a smFRET time trace and the corresponding *Stepfinder* fit (red line). The binding frequencies (total number of binding events/total observation time) were calculated based on *Stepfinder* fits. The total observation time is the sum of individual observation times for each molecule obtained from smFRET time traces. The individual observation time for each molecule is the period from the start of recording to donor photobleaching or to the end of the recording, whichever comes first. Molecules which did not show any binding events were also included in the statistics and contributed to total observation time. To illustrate, seven binding events are observed in the top FRET time trace in Figure 3A within 160 s of observation time. The binding frequency for this molecule is $7/\ ( {160\ {\rm s}} ) = \ 4.38\ \times {10^{ - 2}}\ {{\rm s}^{ - 1}}$. Bootstrapping analysis (54) was used to determine the error bars associated with binding frequencies for each G-Tract construct. In Bootstrapping analysis, a MATLAB script generated 20 000 bootstrapping sets with a 95% confidence level from a set of data that lists the number of binding events in each time trace (including traces that did not show any binding). The total number of binding events in each bootstrapping set was then divided by the total observation time for each set. This results in a distribution of frequencies as shown in Figure 5A, which can be fit to a Gaussian function to determine the peak frequency and standard deviation. The peak positions of these distributions are in excellent agreement with the mean frequencies determined by dividing the total number of binding events with total observation time of the actual dataset (not the generated bootstrapping datasets). The standard deviation of these distributions was used to estimate the uncertainty in the binding frequencies plotted in Figure 5B-C. Table 1 presents the total number of time traces, the total number of binding events, the total observation time, and the resulting binding frequency for each DNA construct.

**Table 1.** Statistics used for binding frequency calculations in Figure 5. *N*_DNA_ refers to number of DNA molecules (time traces) analyzed, *N*_B_ to number of PNA binding events, *T*_tot_ to total observation time, *f*_ave_ to average frequency (*N*_B_/*T*_tot_) and *f*_peak_ to the peak frequency of the bootstrapping distribution in Figure 5A

Construct	*N*_DNA_	*N*_B_	*T*_tot_ (s)	*f*_ave_ ($ \times {10^{ - 3}}{\boldsymbol{\ }}$s^−1^)	*f*_peak_ ($ \times {10^{ - 3}}{\boldsymbol{\ }}$s^−1^)
4G-Tract	2017	331	104353	3.17	3.15
8G-Tract	1632	395	110326	3.58	3.57
12G-Tract	1651	476	92123	5.17	5.16
16G-Tract	2252	557	110646	5.03	5.20
20G-Tract	1748	449	92700	4.84	4.48
24G-Tract	1794	567	89421	6.34	6.33

**Figure 4.**
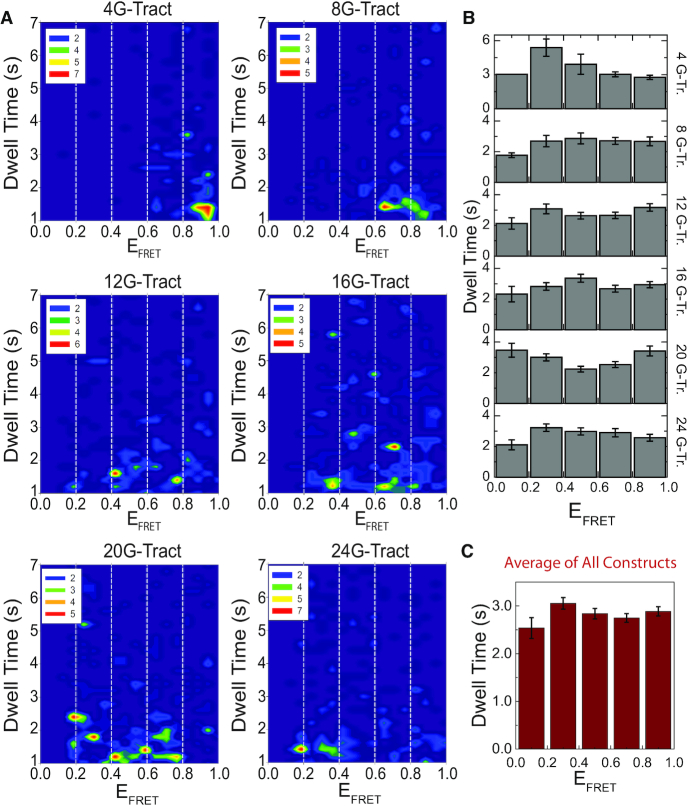
Dwell Time distribution. (**A**) Contour plots of dwell times *vs*. FRET levels. The dwell time range of 1–7 s is shown here while contour plots for shorter dwells are given in Supplementary Figure S4. The number of binding events for 4G-, 8G-, 12G-, 16G-, 20G- and 24G-Tract constructs are *N* = 195, 209, 293, 313, 285 and 323, respectively. As expected, the population of lower FRET states gradually increases with increasing number of G-Tracts. The dashed white lines indicate the five FRET segments, each spanning a FRET range of 0.2, we considered for the analysis in (B) and (C). (**B**) Average dwell times at different FRET segments for each construct. Lower FRET levels are closer to the 3′-end of the DNA construct. **(C)** The dwell times for each FRET range in (B) are averaged over all constructs. The error bars in (B) and (C) are the standard error of the mean for each FRET segment. Statistical analysis did not show significant difference between the dwell times for different FRET segments within each construct in (B) or between the FRET segments of the averaged histogram in (C).

**Figure 5.**
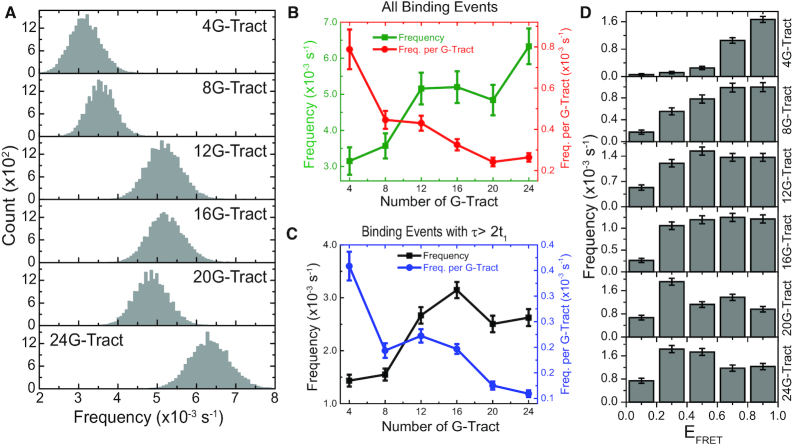
PNA binding frequencies. (**A**) Binding frequency histograms from bootstrapping analysis. The peak value of this distribution represents the average frequency while the spread describes the uncertainty associated with the average. Each histogram was generated from 20 000 different bootstrapping sets. The statistics associated with these data are given in Table 1. (**B**) Scatter plot of average binding frequency (green) and average binding frequency per G-Tract (red) for all events (both short and long dwell times included). The binding frequency tends to increase as the number of G-Tracts (potential binding sites) increases, while the binding frequency per G-Tract consistently decreases. (**C**) Scatter plot of average binding frequency (black) and average binding frequency per G-Tract (blue) for events with longer dwell times (only events with τ>2t_1_ are included). The trends are similar to the case when all binding events were included. The error bars for the frequencies in (B) and (C) are calculated from the standard deviations of the Gaussian fits to the distributions in (A). (**D**) The distribution of average binding frequencies for five FRET segments, each spanning a FRET range of 0.2. Higher FRET states are closer to the 5′-end. Since 4G-Tract and 8G-Tract constructs are relatively short, the binding frequencies are restricted to high FRET levels. The error bars are based on bootstrapping analysis.

Origin software was used for data analysis in Figures 2B, 4B, C and 5D, and for fitting single ($y\ = {y_0}\ + {A_1}{e^{ - [ {( {x - {x_0}} )/{t_3}} ]}}$) and double ($y\ = {y_0}\ + {A_1}{e^{ - [ {( {x - {x_0}} )/{t_1}} ]}} + {A_2}{e^{ - [ {( {x - {x_0}} )/{t_2}} ]}}$) exponential decay functions to the histograms in Figure 3B. Origin was also used for statistical hypothesis testing in Figures 3B, 4B, C and 5D. The number of molecules or number of binding events included in each data set is given in the respective figure caption.

## RESULTS AND DISCUSSION

### FRET-PAINT assay to probe accessibility of long telomeric overhangs

A FRET-PAINT assay was developed to study the accessibility of tandem GQs formed in long telomeric sequences (Figure 1A and B). Binding of an imager strand, Cy5-PNA, to available (unfolded) G-Tracts for a short period of time results in bursts in acceptor intensity and FRET efficiency (*E*_FRET_). Binding to different G-Tracts results in burst with different *E*_FRET_ levels. Figure 1C shows a sample smFRET time trace demonstrating such bursts.

The choice of PNA as the imager strand was crucial for this assay. The dissociation of imager strand should be fast enough to ensure individual binding events to a docking construct do not overlap, which sets an upper limit on imager strand concentration. On the other hand, the concentration should be high enough to observe a large number of binding events and achieve reliable statistics (50). The Cy5-PNA strand concentration was kept at 40 nM, which was high enough to yield reliable binding statistics but low enough to maintain low fluorescence background and negligible false-positive FRET spikes due to non-specific binding of Cy5-PNA to the surface in vicinity of docking constructs. The imager strand should also remain bound to the docking construct for a long-enough time to allow reliable detection of binding events, i.e. at least a few times longer than frame integration time (100 ms for our measurements). This can normally be achieved by using imager strands that are longer and have more complementary bases to the docking construct. However, using longer imager strands will require them to bind to more than one G-Tract, resulting in crosstalk between neighboring G-Tracts. Ideally, the imager strand should have complementarity to only a single G-Tract, e.g. 6 nt or less. Even though different sequences than ours were used, the disassociation times for 6 nt, 7 nt, 8 nt and 9 nt long DNA imager strands were reported as 3.7 ms, 4.8 ms, 63 ms and 670 ms, respectively (50). Given these dissociation times and limitations in time resolution of smFRET assays (10–100 ms), the minimum feasible length of a DNA imager strand would be 8–9 nt. However, our studies demonstrate that dissociation times greater than 1 s are possible with a PNA imager strand that is complementary to a single G-Tract.

Supplementary Figures S2 and S3 demonstrate proof-of-principle measurements that support the use of FRET-PAINT assay to study accessibility of long telomeric overhangs. The presence of GQs and their stability is expected to influence both the binding frequency and dwell time of PNA strands to telomeric overhangs. Higher stability GQs are expected to unfold less frequently, resulting in less frequent PNA binding events. Upon their folding, higher stability GQs are also expected to expel any bound PNA strands more effectively from telomeric overhangs. To test whether these are observed in the FRET-PAINT data, we compared the dwell times and binding frequencies for 12G-Tract and 16G-Tract constructs in 150 mM KCl and 150 mM LiCl. As K^+^ is significantly more effective in stabilizing GQs compared to Li^+^, we expect longer dwell times and higher binding frequencies in LiCl compared to KCl, which were confirmed in the data presented in Supplementary Figures S2B-C and S3. The average binding frequencies for the 12G-Tract and 16G-Tract constructs were 8.4-fold and 6.8-fold, respectively, greater in LiCl compared to KCl (Supplementary Figure S2B-C). The characteristic dwell times (*t*_2_) were 2.1-fold and 2.5-fold longer in LiCl compared to KCl for 12G-Tract and 16G-Tract constructs, respectively (Supplementary Figure S3). In addition, we compared the effect of a GQ stabilizing small molecule (36) on binding frequencies for 12G-Tract and 16G-Tract constructs in KCl (Supplementary Figure S2B, C). For both 12G-Tract and 16G-Tract constructs, the binding frequencies were 2.9 fold lower with the small molecule compared to its absence. Finally, we compared the binding frequencies in KCl and LiCl for a construct that cannot form GQ (1G-Tract construct) and has a single binding site. We observed essentially the same binding frequencies for the 1G-Tract construct in KCl and LiCl (Supplementary Figure S2A), suggesting the differences we observed for GQ forming constructs are due to variations in GQ stability.

Histograms of FRET bursts for 4G-Tract, 8G-Tract, 12G-Tract, 16G-Tract, 20G-Tract and 24G-Tract constructs are shown in Figure 2A. As the number of G-Tracts increases, lower FRET states become more populated, since binding sites further from the donor become available for Cy5-PNA. The increase in population of lower FRET states is quantified by the ratio of the total population of [E_FRET_<0.50] to [E_FRET_>0.50] states, as shown in Figure 2B. With the increase in the number of G-Tracts in the overhang, this ratio increases from 0.13 for 4G-Tract to 0.72 for 24G-Tract. The compaction of the overhang by formation of GQs has made it possible to study such long sequences with FRET.

### Dwell time analysis

The *Stepfinder* program was used to characterize the *E*_FRET_ levels and dwell times (τ) of the binding events in smFRET time traces. Figure 3A shows an smFRET time trace with the *Stepfinder* fit (red line). The dashed line at *E*_FRET_ = 0.13 (*E*_FRET_ = 0.25 before DO correction) indicates the threshold FRET level for considering a FRET burst a Cy5-PNA binding event. This threshold FRET level was determined based on the FRET distributions presented in Figure 2A. As indicated by the red arrow in the 24G-Tract histogram, the longest construct we studied, there is negligible FRET population due to binding events at *E*_FRET_ <0.13. The events were also required to stay above this threshold FRET level for 4 consecutive points, i.e. τ ≥300 ms, to distinguish them from random signal fluctuations. The distributions of dwell times for all constructs are shown in Figure 3B. The dwell time distributions are fitted using single and double exponential decay functions, which are shown as a dashed cyan lines and solid red lines in Figure 3B. The resulting fit parameters of double exponential decay are given in Supplementary Table S2. The double exponential fit was significantly better than the single exponential fit for all constructs (*P* < 0.001 for all constructs). The F-statistics for this comparison are given in Supplementary Table S3. The double exponential fits yielded a fast decay time constant *t_1_* and a slower decay constant *t_2_*, which are listed on the respective histograms in Figure 3B. While the fast decay times are consistent among all constructs (≈0.7 ± 0.1 s), slower decay constants are in the 4.0–8.6 s range, without an obvious dependence on length. The consistency of the short dwell times for different constructs implies they result from the same mechanism, such as partial hybridization of the PNA to the G-Tracts or to the TTA loops in the GQ.

The dwell time data can be further dissected to investigate whether binding of PNA to different segments of a construct, which should yield different FRET levels, results in different dwell times. Contour plots of dwell time *vs*. E_FRET_ were constructed to answer this question (Figure 4A). In order to eliminate short binding events, which may be due to partial hybridization of the PNA probe to the telomeric sequences, we included the dwell times greater than 1.0 s in these contour plots. For clarity of the figures, the upper limit in these plots is set at 7.0 s, even though sparsely populated pockets exist at longer dwell times. The contour plots that include shorter dwell times (<1.0 s) are given in Supplementary Figure S4. Cy5-PNA binding to the 3′-end and 5′-end of the DNA construct yields lower and higher FRET levels, respectively. As in Figure 2A, the population of lower FRET states gradually increases with increasing overhang length. To estimate the average dwell times for different positions of the overhang, the E_FRET_ scale (0.0–1.0) was divided into five FRET segments of equal 0.2 width, as shown with white dashed lines in Figure 4A. For each construct, average dwell times at each of these segments were calculated as shown in Figure 4B. Except for the 20G-Tract construct, a one-way ANOVA analysis did not show a significance difference between the average dwell times for different FRET segments for any of the constructs. To obtain a global average, the dwell times for each FRET range were averaged over all the constructs (Figure 4C). One way ANOVA analysis did not show significant difference in dwell times for different FRET segments in this case either. The one way ANOVA test statistics are given in Supplementary Table S4. Based on these, we conclude that the dwell times for binding to different segments of the telomeric overhang are not distinguishable within the resolution of our measurements.

### PNA binding frequency

The accessibility of telomeres can be quantified by calculating the binding frequency of Cy5-PNA strands to the G-Tracts. The binding frequency is calculated by dividing the total number of binding events by the total observation time for each construct. The number of binding events was determined using a custom MATLAB code that processed the fits generated by *Stepfinder* and counted binding events that have a dwell time >300 ms and *E*_FRET_ >0.13, as described earlier.

Figure 5A shows the distribution of average frequencies obtained by bootstrapping analysis. The peak values of these distributions are in excellent agreement with the calculated average binding frequencies obtained by dividing total number of binding events with the total observation time. The spread in the distributions describes the uncertainty in the average binding frequencies. The error bars in Figure 5B and C are the standard deviation of Gaussian fits to the bootstrapping distributions shown in Figure 5A.

Figure 5B shows that the binding frequency generally increases with increasing telomere length, which is consistent with increasing number of potential binding sites. On the other hand, the binding frequency per G-Tract consistently decreases with telomere length (Figure 5B, where both short and long dwell times are included). Similar trends are also observed when only binding events with τ > 2*t*_1_ are considered, to eliminate events that potentially represent partial hybridization of PNA (Figure 5C). The decrease in the binding frequency per G-Tract with increasing telomere length suggests a compaction in the structure which becomes more prominent as telomere length increases. This might be due to formation of higher order structures, possibly mediated by stacking GQs.

Figure 5D shows the binding frequencies classified based on their FRET levels, where the FRET scale is divided into five segments as in Figure 4B. As expected, due to proximity of the donor fluorophore to the available binding sites in the shorter constructs (such as 4G-Tract or 8G-Tract), higher binding frequencies are observed for higher FRET segments. The binding frequencies are more evenly distributed for longer constructs (such as 20G-Tract or 24G-Tract), since binding sites further from the donor fluorophore become available for PNA binding. To test whether the observed binding frequencies demonstrate significant variations for different FRET segments, a one-way repeated measures ANOVA analysis was performed, which showed significant differences within each construct. The results of this test are given in Supplementary Table S5. This is significant particularly for the longer constructs which show higher binding frequencies at the intermediate segments compared to the 3′ or 5′ ends. We also performed a pairwise comparison test for the FRET segments of 20G-Tract and 24G-Tract construct where the binding frequencies for different segments are compared to each of the other segments. The results of this analysis is presented in the form of contour plot of *t*-values in Supplementary Figure S5 and in tabular form in Supplementary Table S6. These analyses suggest that the GQs at the 3′ and 5′ ends of the overhangs are on average less accessible compared to intermediate regions, which is consistent with GQs inhibiting exonuclease activity at telomeric ends (55).

Our studies demonstrate that even though binding events become more frequent as the length of telomeric DNA is increased, binding frequency per G-Tract consistently decreases with increasing telomere length, suggesting an overall compaction of the structure at longer lengths. This provides a potential framework to understand how longer telomeric overhangs reduce their accessibility by forming more compact structures. This compaction would facilitate protection of telomeric ends against nuclease activity or agents that would otherwise induce DNA damage. It would also make telomeric overhangs less accessible for ssDNA binding proteins, such as RPA, whose accumulation at telomeres would trigger DNA damage checkpoint. On the other hand, a more compact structure might also make telomeric GQs less accessible for small molecules and impose more stringent requirements on potential anti-cancer drugs designed to inhibit telomerase activity.

On technical grounds, we demonstrate the feasibility of using FRET-PAINT as a tool to study accessibility of telomeric sequences similar in length to those encountered in physiological setting. By using PNA as the probe, which forms a more stable hetero-duplex with DNA compared to DNA–DNA homo-duplex or DNA–RNA hetero-duplex, it was possible to reduce the size of the probe such that a single G-Tract is interrogated at a time, while maintaining measurable dwell times (∼1 s). Our studies also demonstrate the feasibility of expanding this approach to more complex settings where the accessibility of telomeres is studied in the presence of Shelterin proteins, DNA binding proteins, helicases, or crowding agents.

## DATA AVAILABILITY

All single molecule FRET data presented in this manuscript can be made available upon request from the corresponding author.

## Supplementary Material

gkab067_Supplemental_File
