# Supplementary material for: Interrogating accessibility of telomeric sequences with FRET-PAINT: evidence for length-dependent telomere compaction
Source: Nucleic Acids Res. 2021 Mar 10;49(6):3371–80. doi: 10.1093/nar/gkab067 (PMC8034622; doi:10.1093/nar/gkab067)
Supplement: gkab067_Supplemental_File [file gkab067_supplemental_file.pdf]

Supplementary Information

Interrogating Accessibility of Telomeric Sequences with FRET-PAINT: Evidence for Length-Dependent Telomere Compaction

Golam Mustafa[1], Sajad Shiekh[1], Keshav GC[2], Sanjaya Abeysirigunawardena[2], and Hamza Balci[1,*]

[1] Department of Physics, Kent State University, Kent, OH 44242, USA;

[2] Department of Chemistry and Biochemistry, Kent State University, Kent, OH 44242, USA; *Correspondence: hbalci@kent.edu; Tel.: +1-330-672-2577

Purification of DNA Constructs

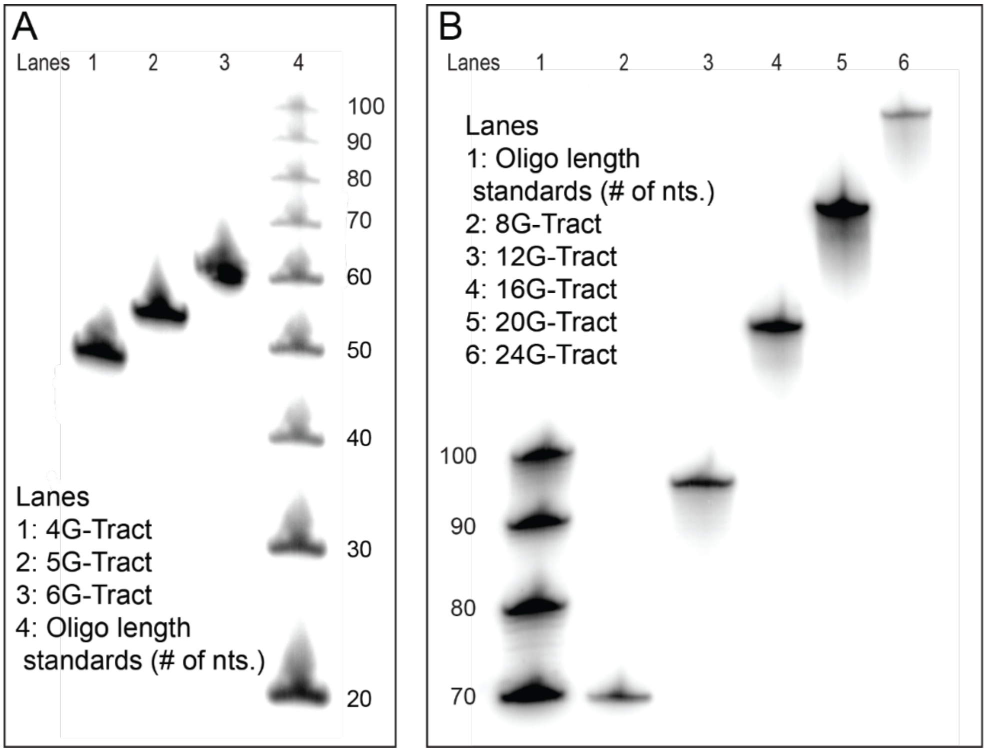

Figure S1: Radiograph of denaturing polyacrylamide gel that shows purified DNA oligonucleotides radiolabeled with ^{32}P isotope. **(A)** 10% denaturing polyacrylamide gel. The lanes 1, 2, 3, and 4 contain 4, 5, 6G-Tract DNA oligonucleotides, and oligo standards, respectively. The numbers next to lane 4 represent the number of nucleotides in the oligo length standards. **(B)** 8% denaturing polyacrylamide gel. Lanes 1, 2, 3, 4, 5, and 6 contain oligo standards, 8, 12, 16, 20, and 24G-Tract DNA oligonucleotides, respectively. The numbers before lane 1 represent the number of nucleotides in the oligo standards.

Impact of Ions and Small Molecules on Binding Frequency

For G-quadruplex forming sequences, the binding frequency of PNA strands systematically decreases as the stability of GQ is increased. The binding frequency in KCl is significantly lower than that in LiCl, in which the GQ is less stable. Including L1H1-7OTD (referred to as 7OTD in the figure), which is a GQ stabilizing small molecule, further decreases the binding frequency. We also measured the binding frequency of PNA for a 1G-Tract construct (see Table S1 for sequence), which contains a single GGGTTA sequence and is incapable of forming a GQ. In this case, the frequency is essentially the same in KCl and LiCl, suggesting the differences observed in GQ-forming constructs are due to the change in GQ stability in the presence of different ions.

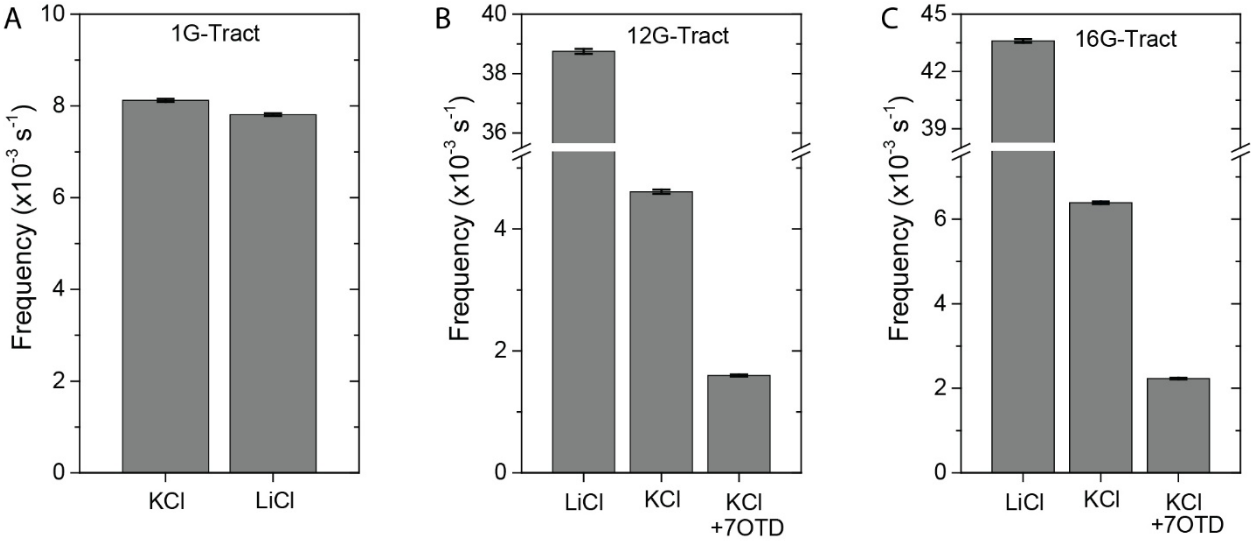

Figure S2: (A) The average binding frequency of PNA to the 1G-Tract construct, which contains a single binding site, is very similar in KCl and LiCl. This construct does not form any secondary structures. The binding frequency for KCl and LiCl are (8.1±0.1) $\times10^{-3}$ s^{-1} and (7.8±0.1) $\times10^{-3}$ s^{-1}, respectively. **(B)** and **(C)** show binding frequencies for 12G-Tract and 16G-Tract constructs, respectively, in 150 mM LiCl, 150 mM KCl, and 150 mM KCl+1 μM L1H1-7OTD. For both constructs, the binding frequency is highest in LiCl, which is the weakest stabilizer of GQ and lowest in KCl+7OTD, in which GQ is most stable. The binding frequencies for the 12G-Tract construct are as follows: (38.8±0.1) $\times10^{-3}$ s^{-1} in LiCl, (4.6±0.1) $\times10^{-3}$ s^{-1} in KCl, and (1.6±0.1) $\times10^{-3}$ s^{-1} in KCl+7OTD. The binding frequencies for the 16G-Tract construct are as follows: (43.6±0.1) $\times10^{-3}$ s^{-1} in LiCl, (6.4±0.1) $\times10^{-3}$ s^{-1} in KCl, and (2.2±0.1) $\times10^{-3}$ s^{-1} in KCl+7OTD. The error bars are standard error of the mean.

Dwell Time in LiCl

The dwell time in LiCl is significantly longer than that in KCl for 12G-Tract and 16G-Tract constructs. The dwell times in KCl are reported in Fig. 3 as t_2=5.5±0.4 s for 12G-Tract, and t_2=4.3±0.2 s for 16 G-Tract. The corresponding values in LiCl are: t_2=11.4±1.2 s for 12G-Tract, and t_2=10.6±0.6 s for 16G-Tract.

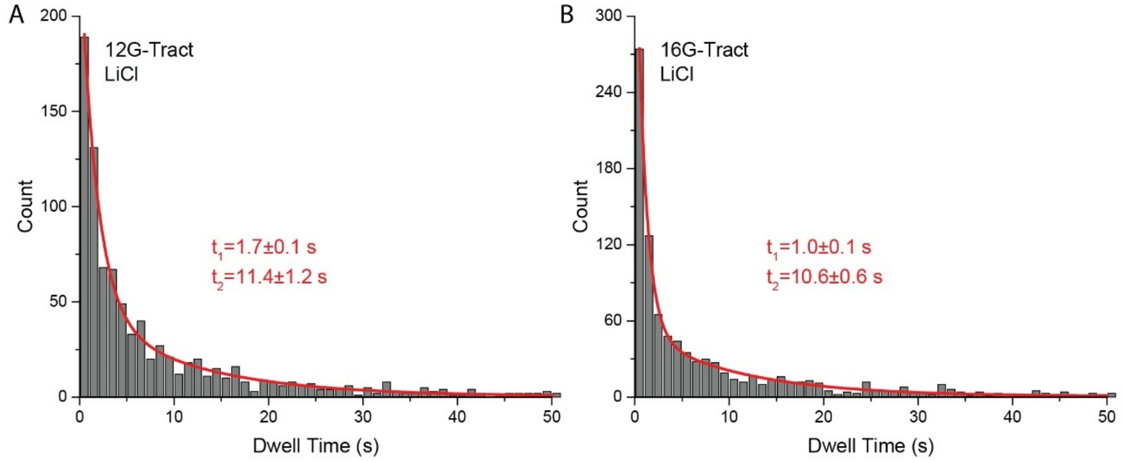

Figure S3: (A) The dwell time for 12G-Tract in 150 mM LiCl. **(B)** The dwell time for 16G-Tract in 150 mM LiCl.

Dwell Time Distributions in 0-2 s Range

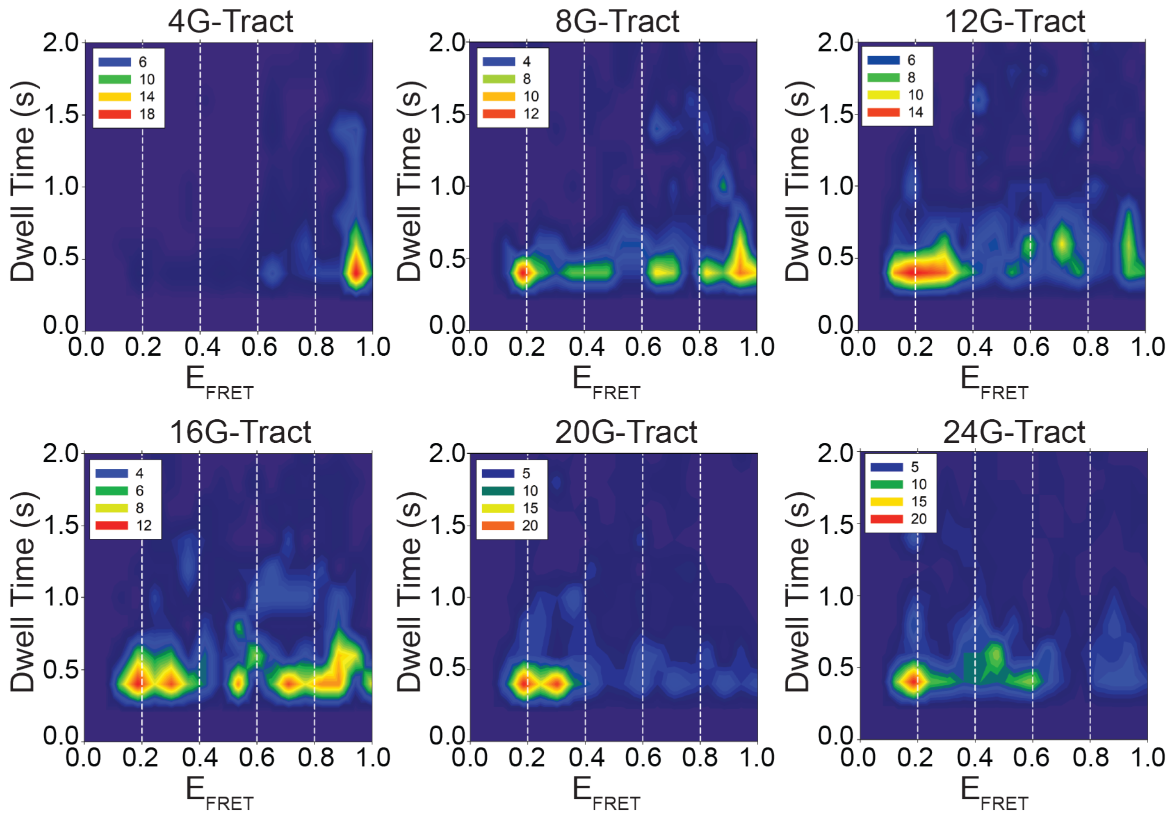

Figure S4: Contour plots of dwell time *vs.* E_{FRET} for dwell times in the 0-2 s range, which includes the shorter dwell times that were not shown in Fig. 4A. The number of binding events for 4G-Tract, 8G-Tract, 12G-Tract, 16G-Tract, 20G-Tract, and 24-Tract are 331, 394, 561, 556, 563 and 604, respectively.

Statistical Analysis of Binding Frequencies for Different Segments of the Construct

Fig. S5 shows contour plots of t-values for the longer constructs (20G-Tract and 24G-Tract) that have significant numbers of binding events at both low and high FRET efficiencies. This analysis was performed to investigate whether binding frequencies show significant differences when they are grouped based on their corresponding FRET values. High-FRET efficiencies ($0.8<E_{FRET}\leq 1.0$) correspond to binding to the vicinity of 5'-end while low FRET efficiencies ($0.0<E_{FRET}\leq 0.2$) correspond to binding to the vicinity of 3'-end. The intermediate FRET values ($0.2<E_{FRET}\leq 0.8$) represent binding to the regions between these two. In this analysis, the frequencies within each of these FRET segments are compared with the frequencies in the other segments in a pairwise manner. Larger t-values indicate larger differences between binding frequencies for the segments that are compared. Such comparisons are particularly meaningful for longer constructs, such as 20G-Tract and 24G-Tract, for which all FRET segments have a significant number of binding events throughout the FRET range. For shorter constructs, lower FRET segments have very few binding events (due to proximity of donor-acceptor fluorophores), reducing the statistical significance of this comparison. The 20G-Tract and 24G-Tract constructs showed high t-values in comparisons between low (0.0-0.2) and intermediate (0.2-0.4) FRET segments. They also showed high t-values in comparisons between high (0.8-1.0) and intermediate (0.2-0.4) FRET segments. Comparatively, the t-values were smaller when low (0.0-0.2) and high (0.8-1.0) FRET segments were compared. These t-values are given in tabular form in Table S6 as contour plots in Fig. S5.

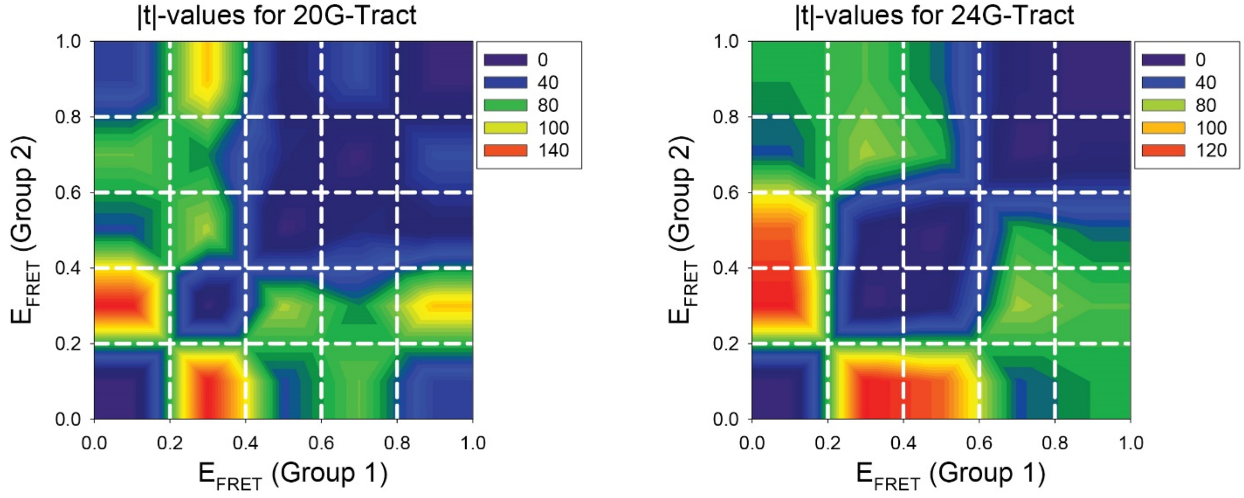

Figure S5: The t-values based on pairwise comparisons of the binding frequencies for different FRET segments for 20G-Tract and 24G-Tract constructs. One of the comparison groups is plotted along the x-axis while the other along the y-axis. For example, the t-value at the intersection of (0.4-0.6) bin along x-axis and the (0.0-0.2) bin along the y-axis represents the t-value for the comparison of binding frequencies for events that fall within these FRET segments. Large t-values indicate more significant differences between the frequencies of comparison groups. For the longest constructs 20G-Tract and 24G-Tract, the largest differences are observed when the two ends (0.0-0.2) and (0.8-1.0) are compared with the intermediate ranges (0.2-0.8).

Table S1: Sequences of DNA and PNA strands which were used in the study. The partial duplex DNA constructs were created by annealing the stem strand (18 nt) with a longer strand that includes the G-Tracts. The nucleotides in green fonts constitute the overhang. The subscripts designate the number of repeats, i.e. (GGGTTA)$_{8}$ refers to the sequence: GGGTTAGGGTTAGGGTTAGGGTTAGGGTTAGGGTTAGGGTTAGGGTTA.

The red nucleotides of Cy5-PNA strand are complementary to a single G-tract.

**Strand**	**Sequence** (5′–3′)
1G-Tract	TGGCGACGGCAGCGAGGCTTTTTTTTTTTTAGGGTTA
4G-Tract	TGGCGACGGCAGCGAGGCTTAGGGTTAGGGTTAGGGTTAGGGTT
8G-Tract	TGGCGACGGCAGCGAGGCTT(GGGTTA)$_{8}$
12G-Tract	TGGCGACGGCAGCGAGGCTT(GGGTTA)$_{12}$
16G-Tract	TGGCGACGGCAGCGAGGCTT(GGGTTA)$_{16}$
20G-Tract	TGGCGACGGCAGCGAGGCTT(GGGTTA)$_{20}$
24G-Tract	TGGCGACGGCAGCGAGGCTT(GGGTTA)$_{24}$
Stem	Cy3-GCCTCGCTGCCGTCGCCA-Biotin
Cy5-PNA	TAACCCTT-Cy5

Table S2: The resulting fit parameters of double exponential decay functions of Figure 3B.

$$y = y_0 + A_1 e^{-[(x-x_0)/t_1]} + A_2 e^{-[(x-x_0)/t_2]}$$

DNA Construct	y_0	x_0	A_1	t_1	A_1	t_2
4G-Tract	0.13±0.13	0.44	100.62	0.77±0.06	43.51	3.96±0.33
8G-Tract	0.08±0.05	0.16	274.08	0.75±0.01	16.46	8.57±0.49
12G-Tract	0.39±0.12	0.20	358.11	0.71±0.02	36.12	5.54±0.40
16G-Tract	0.40±0.11	-0.02	424.28	0.63±0.02	63.68	4.32±0.21
20G-Tract	0.25±0.10	0.24	329.46	072±0.02	49.77	4.28±0.26
24G-Tract	0.24±0.27	0.29	324.75	0.86±0.03	27.68	7.41±1.03

Table S3: F-Statistics for comparing single and double exponential decay functions for the data in Figure 3B. The F-statistics are reported as:
F(Numerator degrees of freedom, Denominator degrees of freedom)=Calculated F value, p=p-value.

**DNA Construct**	**F-Statistics**
4G-Tract	F(2,81)=146.59, p=.001
8G-Tract	F(2,173)= 567.88, p=.001
12G-Tract	F(2,109)= 330.82, p=.001
16G-Tract	F(2,114)= 587.22, p=.001
20G-Tract	F(2,123)= 411.51, p=.001
24G-Tract	F(2,83)= 99.89, p=.001

Table S4: One-way ANOVA analysis results comparing the dwell times for different FRET segments within each construct (Fig. 4B). Similarly, one-way ANOVA analysis was performed to compare the dwell times for different FRET segments of the averaged distribution (Fig. 4C). The one-way ANOVA test results are reported in the same format as in Table S3. Except the 20G-Tract construct, $p>0.05$ for all constructs and the averaged distribution, suggesting there is no statistically significant difference between the dwell times of different FRET segments.

DNA Construct	ANOVA Test
4G-Tract	$F(4, 148)=2.28$, $p=.063$
8G-Tract	$F(4, 121)=0.22$, $p=.929$
12G-Tract	$F(4, 191)=1.61$, $p=.174$
16G-Tract	$F(4, 208)=1.30$, $p=.271$
20G-Tract	$F(4, 211)=4.05$, $p=.003$
24G-Tract	$F(4, 225)=2.08$. $p=.085$
All Constructs	$F(4, 1129)=1.58$, $p=.177$

Table S5: Repeated measures ANOVA Test Statistics comparing the binding frequencies for different FRET segments of Fig. 5D. The repeated measures ANOVA test results are reported in the same format as in Table S3. The binding frequencies for different segments were significantly different from each other ($p=.001$) for all constructs.

DNA Construct	Pillai's Trace Value	ANOVA test
4G-Tract	0.999	$F(4, 195)= 47310.08$, $p=.001$
8G-Tract	0.995	$F(4, 195)= 9398.95$, $p=.001$
12G-Tract	0.990	$F(4, 195)= 4846.68$, $p=.001$
16G-Tract	0.996	$F(4, 195)= 12326.15$, $p=.001$
20G-Tract	0.987	$F(4, 195)= 3833.33$, $p=.001$
24G-Tract	0.987	$F(4, 195)= 3780.65$, $p=.001$

Table S6: The t-values of the pairwise comparison of the binding frequencies for different FRET segments of Figure 5D for 20G-Tract and 24G-Tract constructs. These data are plotted as contour plots in Figure S5.

DNA Construct	FRET segments pair	DF	|t|value	Prob>|t|
20G-Tract	0.0-0.2 vs. 0.2-0.4	792	152.16	0.001
	0.0-0.2 vs. 0.4-0.6	792	57.92	0.001
	0.0-0.2 vs. 0.6-0.8	792	84.28	0.001
	0.0-0.2 vs. 0.8-1.0	792	38.51	0.001
	0.2-0.4 vs. 0.4-0.6	792	94.24	0.001
	0.2-0.4 vs. 0.6-0.8	792	67.89	0.001
	0.2-0.4 vs. 0.8-1.0	792	113.65	0.001
	0.4-0.6 vs. 0.6-0.8	792	26.35	0.001
	0.4-0.6 vs. 0.8-1.0	792	19.41	0.001
	0.6-0.8 vs. 0.8-1.0	792	45.77	0.001
24G-Tract	0.0-0.2 vs. 0.2-0.4	792	128.63	0.001
	0.0-0.2 vs. 0.4-0.6	792	114.02	0.001
	0.0-0.2 vs. 0.6-0.8	792	50.75	0.001
	0.0-0.2 vs. 0.8-1.0	792	60.29	0.001
	0.2-0.4 vs. 0.4-0.6	792	14.60	0.001
	0.2-0.4 vs. 0.6-0.8	792	77.87	0.001
	0.2-0.4 vs. 0.8-1.0	792	68.34	0.001
	0.4-0.6 vs. 0.6-0.8	792	63.27	0.001
	0.4-0.6 vs. 0.8-1.0	792	53.73	0.001
	0.6-0.8 vs. 0.8-1.0	792	9.53	0.001
